# Impact of Coronary Artery Disease and Diabetes Mellitus on the Long-Term Follow-Up in Patients after Retrograde Recanalization of the Femoropopliteal Arterial Region

**DOI:** 10.1155/2019/6036359

**Published:** 2019-03-31

**Authors:** Joanna Wojtasik-Bakalarz, Zoltan Ruzsa, Tomasz Rakowski, Andreas Nyerges, Krzysztof Bartuś, Agata Stanek, Dariusz Dudek, Andrzej Surdacki, Paweł Kleczyński, Stanisław Bartuś

**Affiliations:** ^1^2nd Department of Cardiology, Jagiellonian University Collegium Medicum, Krakow, Poland; ^2^Semmelweis University, Heart and Vascular Center, Cardiology Department, Hungary; ^3^Bács-Kiskun County Hospital, Invasive Cardiology Department, Teaching Hospital of Szent-Györgyi Albert Medical University, Kecskemét, Hungary; ^4^Department of Internal Medicine, Angiology and Physical Medicine, School of Medicine with the Division of Dentistry in Zabrze, Medical University of Silesia, Bytom, Poland

## Abstract

The most relevant comorbidities in patients with peripheral artery disease (PAD) are coronary artery disease (CAD) and diabetes mellitus (DM). However, data of long-term follow-up of patients with chronic total occlusion (CTO) are scarce. The aim of the study was to assess the impact of CAD and DM on long-term follow-up patients after superficial femoral artery (SFA) CTO retrograde recanalization. In this study, eighty-six patients with PAD with diagnosed CTO in the femoropopliteal region and at least one unsuccessful attempt of antegrade recanalization were enrolled in 2 clinical centers. Mean time of follow-up in all patients was 47.5 months (±40 months). Patients were divided into two groups depending on the presence of CAD (CAD group: *n* = 45 vs. non-CAD group: *n* = 41) and DM (DM group: *n* = 50 vs. non-DM group: *n* = 36). In long-term follow-up, major adverse peripheral events (MAPE) occurred in 66.6% of patients with CAD vs. 36.5% of patients without CAD and in 50% of patients with DM vs. 55% of non-DM subjects. There were no statistical differences in peripheral endpoints in both groups. However, there was a statistically significant difference in all-cause mortality: in the DM group, there were 6 deaths (12%) (*P* value = 0.038). To conclude, patients after retrograde recanalization, with coexisting CTO and DM, are at higher risk of death in long-term follow-up.

## 1. Introduction

Coexistence of peripheral artery disease (PAD), coronary artery disease (CAD), and cerebrovascular disease causes almost 2 million deaths in Europe each year [1]. There are an increasing number of patients with PAD, who have increased mortality rate and higher risk of cardiovascular events; however, PAD is still underdiagnosed [2]. It is worth pointing out that PAD is an independent risk factor for increased cardiovascular mortality [1]. The most relevant comorbidities in patients with PAD are CAD and diabetes mellitus (DM) [3]. Patients with diagnosed DM are characterized by higher frequency of PAD occurrence [4], and DM itself accelerates the progression of atherosclerosis. Recent studies have shown the impact of glycemia control and severity of CAD on patency of peripheral arteries in patients with PAD and poorer outcomes in long-term follow-up [5]. Chronic total occlusion (CTO) affects around 50% of patients with PAD. Patients with CTO in the femoropopliteal region require complex treatment of PAD and comorbidities [1]. The most challenging cases in patients with CTO are those with unsuccessful antegrade recanalization (around 10-15%) [6]. Data of long-term follow-up of patients with CTO are scarce. The aim of the study was to assess the influence of CAD and DM on the long-term follow-up in patients after superficial femoral artery (SFA) CTO retrograde recanalization.

## 2. Material and Methods

We included 86 patients, enrolled in two centers, with symptomatic PAD and diagnosed chronic total occlusion in the artery of the lower extremity, after at least one attempt of unsuccessful antegrade recanalization. CTO lesions were verified in angiography of the lower extremities' vessels. Patients were screened for most relevant comorbidities and risk factors. Before the procedure, all patients were evaluated according to the Rutherford/Fontaine scale and the ankle-brachial index (ABI) measurements were performed. All patients were qualified for retrograde recanalization, after previous unsuccessful antegrade crossing, and registry description was previously published [7].

The procedure of retrograde recanalization was performed according to the local protocol. Puncture was performed under the guidance of ultrasound or fluoroscopy. All patients required 2 approaches: proximal, mostly in the femoral common artery, and distal, in the distal part of SFA or the proximal part of the popliteal artery. A few patients required procedures also in arteries below the knee.

After the index procedure, patients were administrated dual antiplatelet therapy (75 mg of aspirin, 75 mg of clopidogrel) for 1-3 months, low molecular weight heparin for 1 month, and high dose of statin.

Two separated analyses were performed based on the presence of CAD and DM. Patients were divided into 2 groups: patients with diagnosed CAD and without CAD. CAD was defined as previous acute coronary syndrome, history of percutaneous coronary intervention, bypass grafting (CABG), or lesions in coronary arteries ≥ 50% stenosis evaluated in coronary angiography.

In the second analysis, patients were divided into 2 groups based on the presence of DM—patients with diagnosed DM and without DM. Patients were enrolled to the DM group regardless of the type of treatment (diet, oral medications, and insulin) and duration of DM. Patients with newly diagnosed DM during index hospitalization were excluded from analysis.

All patients were screened during clinical follow-up for major adverse cardiac and cardiovascular events (MACCE), major adverse peripheral events (MAPE), and all-cause mortality.

MACCE was defined as acute coronary artery syndrome, coronary artery intervention, coronary bypass grafting, and stroke/transient ischemic attack (TIA).

MAPE was defined as target vessel reintervention, nontarget vessel reintervention, and amputation.

All patients were screened for all-cause deaths: cardiac and noncardiac.

Regression analysis was performed to find independent predictors for cardiac events, cerebrovascular events, peripheral events, and all-cause death in patients after CTO retrograde recanalization in the femoropopliteal region.

### 2.1. Statistical Analysis

Results are presented as the number of patients (percentage) or mean value ± standard deviation (SD)/median value or interquartile range (IQR) where applicable. Differences between groups were tested using the Chi-square test and Fisher's exact test for dichotomous variables and the Mann-Whitney *U* test for continuous variables. The Kaplan-Meier method was used to assess the difference in mortality during follow-up between patients. Additionally, univariate Cox regression analysis was performed. In all tests, *P* value of <0.05 was considered statistically significant.

## 3. Results

Mean time of long-term follow-up in all patients was 47.5 months (±40 months). Patients were divided into two groups depending on the presence of CAD (CAD: *n* = 45 vs. non-CAD: *n* = 41) and DM (DM: *n* = 50 vs. non-DM: *n* = 36). In group DM vs. non-DM, the average age was similar. In both groups, most of the patients were male. Diabetic patients were treated with insulin, oral hypoglycemic medications, or diet only. Details of medical history are presented in Table 1. In long-term follow-up, MAPE was observed in 50% of patients with DM and 55.5% of patients without DM.

**Figure 1:**
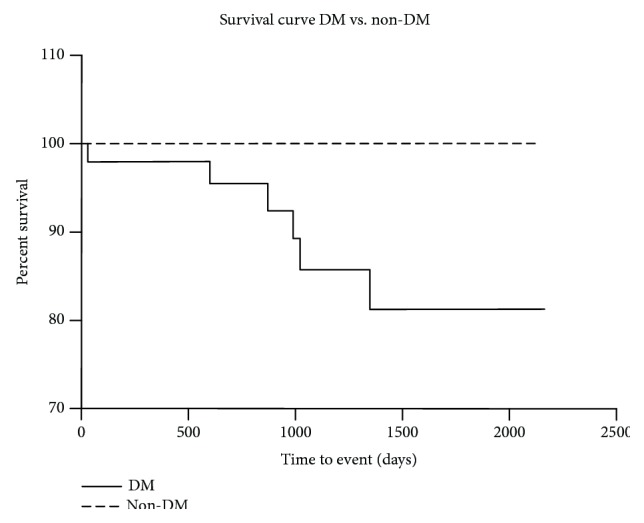
Kaplan-Meier survival curve in group DM vs. non-DM. DM: diabetes mellitus.

Target vessel revascularization, nontarget vessel revascularization, stroke, acute coronary syndrome, and unsuccessful revascularization were similar in both groups (Table 2). The observed number of amputations in the DM group was twofold higher than that in the group without DM, but it was not statistically significant. The all-cause mortality rate was higher in the DM group (12% vs. none, *P* = 0.038, Figure 1). In Cox regression analysis, DM was an independent risk factor for death (*P* = 0.0147).

In the CAD/no-CAD cohort, the number of patients in both groups was similar. The mean age of patients were 66.7 (±11.8) in the CAD group and 75 years (±14.1) in the non-CAD group. Both groups were comparable in terms of the presence of comorbidities. All demographics and medical history are presented in Table 1.

**Figure 2:**
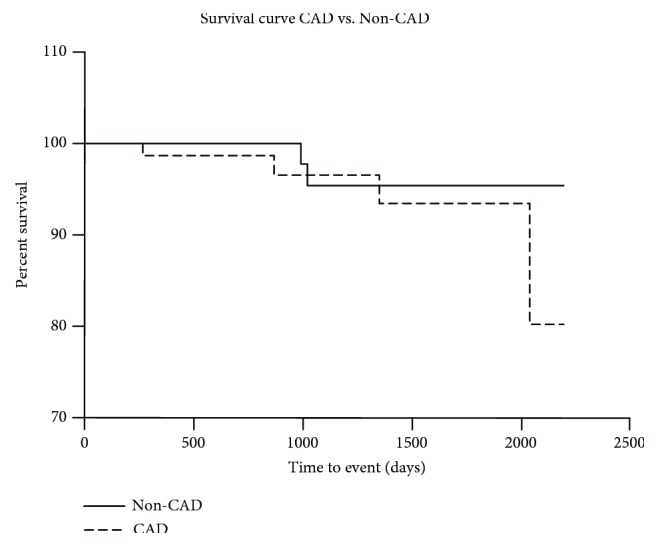
Kaplan-Meier survival curve in group CAD vs. non-CAD. CAD: coronary artery disease.

In long-term follow-up, reinterventions in the target vessel were observed in 27% of the CAD group and in 6% of the non-CAD group. All endpoints are presented in Table 2. MAPE occurred in 66.6% of patients with CAD and in 36.5% of patients without CAD. There was no significant difference in all endpoints in both groups (Table 3 and Figure 2).

## 4. Discussion

PAD is an atherosclerotic disease and becomes an increasing problem in elderly population. PAD alone is associated with a higher rate of mortality and morbidity, including cerebrovascular events, as compared to the mortality and morbidity rate of patients without PAD [2, 8, 9]. Moreover, PAD shares similar risk factors with CAD [10, 11] and patients with comorbid PAD and CAD reach worse outcomes in follow-up than patients with CAD alone [12, 13].

Around 50% of patients with diagnosed symptomatic PAD suffer from CTO [6]. Despite the development of numerous endovascular techniques, CTO lesions in the femoropopliteal region are still challenging for endovascular treatment and those patients required complex treatment. Retrograde recanalization of SFA CTO in patients with at least one unsuccessful antegrade crossing is a safe and effective option with a patency rate at 88.2% after one year [14] and an amputation rate 4.7% in long-term follow-up [7]. The mortality rate in follow-up varies between 6.9% and 23% [7, 15], and the patency rate of the target vessel in critical limb ischemia is up to 42% [16]. However, additional data from long-term follow-up after retrograde recanalization is still limited.

In our study, we confirmed that DM has a relevant impact on long-term outcomes in patients after retrograde revascularization of the femoropopliteal region. Cooccurrence of PAD and DM reaches more than 30% [17]. In patients with coexisting PAD and DM, there is greater severity of PAD, which can be explained by the higher rate of amputation in this population (DM 41.4% vs. non-DM 11.5%) [18]. This trend can be seen also in our study, where the amputation rate was twice lower in the non-DM group. Additionally, the mortality rate in DM patients is twice higher (51.7% vs. 25.6%) than that in non-DM patients [18]. These findings were confirmed by Reiber et al. who pointed out that 5-year mortality in diabetic patients with PAD reaches 50% and overall amputation risk is up to 20% [19]. Our results stay in accordance with previously published data where the mortality rate among patients with coexisting PAD and DM is higher and statistically relevant (12% vs. 0, *P* value = 0.038). Moreover, Jude et al. reported that in a diabetic deceased group of patients, there was a trend toward higher prevalence of CTO in arteries [18].

According to the study by Leibson et al., coexistence of PAD and DM increases risk of death 1.67 times compared to PAD alone and 1.55 times compared to DM alone [2].

On the other hand, Weis-Müller et al. suggested that at 1-, 3-, and 5-year of follow-up, limb salvage rates were not influenced by DM or CAD and 1-, 3-, and 5-year survival was uninfluenced by DM alone, though CAD reduced life expectancy [20].

In patients with PAD, the prevalence of CAD reaches between 46 and 71% [21, 22].

Previous studies point out that PAD is associated with higher rate of cardiovascular adverse events (5.35% vs. 4.52%) [23] and higher rate of total mortality in long-term observation (47.8% vs. 36.4%) than CAD [13]. In analysis of patients who underwent vascular surgery, postoperative and long-term outcomes are determined by CAD [24, 25]. In a group of patients with lower limb reconstruction procedures, the main causes of 30-day mortality were cardiovascular events and infections [25]. Results from our study show higher frequency of amputations and mortality rate in the group of patients with diagnosed CAD. It is also worth pointing out that patients with CAD are characterized by a twofold rise in the reintervention rate in the previous treated lesion. Although results are not statistically significant, it shows a trend of increased tendency of adverse events, including MAPE, in long-term follow-up.

What is more, recent studies show comparison differences between reducing risk factors in patients with CAD and PAD. Patients with CAD are more intensively treated for atherosclerotic risk factors than PAD patients [25, 26]. Administration of hypolipemic drugs was higher in patients with hypercholesterolemia and CAD than in patients with high cholesterol level and PAD (58% vs. 46%) [26].

Our study confirmed that most of the results from studies designed for the general population of patients with PAD can be extrapolated to a subgroup of patients after retrograde recanalization of the femoropopliteal region. Coexistence of DM in those patients is correlated with higher all-cause mortality rate in long-term follow-up, although the amputation rate and TVR revascularization were uninfluenced by DM or CAD. However, the expected impact of CAD on MACCE and MAPE was not confirmed in this study. The study group included a small number of selected patients with CTO and unsuccessful antegrade recanalization. Patients with CAD were on average 10 years younger than the non-CAD group, which could have influenced the results. What is more, the study was planned as a retrospective registry which may be considered limitation; for example, data about duration of DM and level of glycemia control were not collected.

## 5. Conclusion

Patients, after retrograde femoropopliteal recanalization, with coexisting CTO and DM, are at higher risk of death than non-DM patients. Despite our previous study, we do not confirm the impact of CAD on the long-term follow-up in patients with PAD. However, complex treatment of those patients should be focused on intensively reducing atherosclerotic risk factors.

## Figures and Tables

**Table 1:** Demographics and medical history of patients in the DM/non-DM group and CAD/non-CAD group.

	All patients (*n* = 86)	DM (*n* = 50)	Non-DM (*n* = 36)	*P* value	CAD (*n* = 45)	Non-CAD (*n* = 41)	*P* value
Mean age, years (±SD)	65 (±9)	65 (±8.9)	62 (±10.1)	0.57	67 (±11.8)	75 (±14.1)	0.19
Sex (male, *n*, %)	58 (67.4)	33 (60)	25 (69)	0.51	28 (62)	29 (70)	0.39
BMI (kg/m^2^) (±SD)	29 (±5.9)	31	26	0.4	28	30	0.51
Hypertension (*n*, %)	75 (87)	45 (90)	30 (83)	0.55	40 (88.8)	35 (85)	0.64
DM (*n*, %)	50 (58.1)						0.8
(i) Insulin		21 (42)			12 (26.6)	10 (24.4)	0.9
(ii) Oral		28 (56)			14 (31)	13 (31)	0.95
(iii) Diet only		1 (2)			1 (2)	0	
(iv) Non-DM		0	36 (100)		18 (40)	18 (43.9)	0.87
CAD (*n*, %)	45 (52.3)	28 (56)	17 (47)	0.56	45 (100)	0	
COPD (*n*, %)	11 (12.8)	7 (14)	4 (11)	0.3	7 (15.5)	4 (9)	0.42
Hypercholesterolemia (*n*, %)	52 (60.5)	27 (54)	25 (69)	0.1	27 (60)	25 (62)	0.79
Previous stroke/TIA (*n*, %)	8 (9.3)	5 (10)	3 (8)	0.85	4 (8)	4 (9)	0.89
Smoking (*n*, %)	38 (44.2)	21 (42)	17 (47)	0.54	18 (40)	20 (48.7)	0.24

BMI: body mass index; CAD: coronary artery disease; COPD: chronic obstructive pulmonary disease; DM: diabetes mellitus; SD: standard deviation: TIA: transient ischemic attack.

**Table 2:** Major adverse cardiac and cerebrovascular events (MACCE) and other events after SFA CTO revascularization stratified by DM presence.

	All patients (*n* = 86)	DM (*n* = 50)	Non-DM (*n* = 36)	*P* value
TVR (*n*, %)	18 (20.9)	9 (18)	9 (25)	0.4
Non-TVR (*n*, %)	23 (26.7)	13 (26)	10 (27.8)	0.8
Amputation (*n*, %)	4 (4.6)	3 (6)	1 (2.8)	0.8
Stroke (*n*, %)	0	0	0	
ACS (*n*, %)	3 (3.5)	2 (4)	1 (2.8)	0.8
All-cause mortality (*n*, %)	6 (6.9)	6 (12)	0	0.038
Unsuccessful revascularization (*n*, %)	5 (5.8)	2 (4)	3 (8.3)	0.6

ACS: acute coronary syndrome; CTO: chronic total occlusion; SFA: superficial femoral artery; non-TVR: nontarget vessel revascularization; TVR: target vessel revascularization.

**Table 3:** Major adverse cardiac and cerebrovascular events (MACCE) and other events after SFA CTO recanalization stratified by CAD presence.

	All patients (*n* = 86)	CAD (*n* = 45)	Non-CAD (*n* = 41)	*P* value
TVR (*n*,%)	18 (20.9)	12 (27)	6 (14.6)	0.2
Non-TVR (*n*,%)	23 (26.7)	15 (33)	8 (19.5)	0.2
Amputation (*n*,%)	4 (4.6)	3 (6.6)	1 (2.4)	0.36
Stroke (*n*,%)	0	0	0	
ASC (*n*,%)	3 (3.5)	1 (2.2)	2 (4.9)	0.6
All-cause mortality (*n*,%)	6 (6.9)	4 (8.9)	2 (4.9)	0.68
Unsuccessful revascularization (*n*,%)	5 (5.8)	2 (4.4)	3 (7.3)	0.7

ACS: acute coronary syndrome; CTO: chronic total occlusion; SFA: superficial femoral artery; non-TVR: nontarget vessel revascularization; TVR: target vessel revascularization.

## Data Availability

All external data used in this paper are listed in References.
